# Regorafenib Combined with BRAF/MEK Inhibitors for the Treatment of Refractory Melanoma Brain Metastases

**DOI:** 10.3390/cancers16234083

**Published:** 2024-12-05

**Authors:** Iris Dirven, Eden Pierre, An-Sofie Vander Mijnsbrugge, Manon Vounckx, Jolien I. Kessels, Bart Neyns

**Affiliations:** Team Laboratory for Medical and Molecular Oncology (LMMO), Translational Oncology Research Center (TORC), Vrije Universiteit Brussel (VUB), Universitair Ziekenhuis Brussel (UZ Brussel), Laarbeeklaan 101, 1090 Brussels, Belgium

**Keywords:** stage IV-M1d melanoma, brain metastases, regorafenib, BRAF/MEK inhibitors, *BRAF*, *NRAS*, targeted therapy, RAF dimer inhibitor, class II RAF inhibitor

## Abstract

Background: There are no active treatment options for patients with progressive melanoma brain metastases (MBM) failing immune checkpoint blockade (ICB) and BRAF/MEK inhibitors (BRAF/MEKi). Regorafenib (REGO), an oral multi-kinase inhibitor (incl. RAF-dimer inhibition), can overcome adaptive resistance to BRAF/MEKi in preclinical models. Methods: This is a single-center retrospective case series of patients with refractory MBM treated with REGO plus BRAF/MEKi (compassionate use). Results: A total of 22 patients were identified (18 *BRAF*-mutant, 4 *NRAS*^Q61^-mutant; 19 with progressive MBM; 11 on corticosteroids). Thirteen *BRAF*^V600^-mutant patients were progressing on BRAF/MEKi at the time of REGO association. *BRAF*-mutant patients received REGO (40–80 mg once daily) combined with BRAF/MEKi, *NRAS*-mutant patients were treated with REGO + MEKi (+low-dose BRAFi to mitigate skin-toxicity). Grade 3 TRAE included arterial hypertension (*n* = 4) and maculopapular rash (*n =* 3). There were no G4/5 TRAE. In *BRAF*-mutant patients, overall and intracranial objective response rates (overall ORR and IC-ORR) were 11 and 29%, and overall and intracranial disease control rates (overall DCR and IC-DCR) were 44 and 59%, respectively. In *NRAS*-mutant patients overall ORR and IC-ORR were 0 and 25% and overall DCR and IC-DCR were 25 and 50%, respectively. The median PFS and OS were, respectively, 7.1 and 16.4 weeks in *BRAF*-mutant and 8.6 and 10.1 weeks in *NRAS*-mutant patients. Conclusions: In heavily pretreated patients with refractory MBM, REGO combined with BRAF/MEKi demonstrated promising anti-tumor activity with an acceptable safety profile. In *BRAF*^V600^-mutant melanoma patients, responses cannot solely be attributed to BRAF/MEKi rechallenge. Further investigation in a prospective trial is ongoing to increase understanding of the efficacy.

## Simple Summary

In heavily pretreated melanoma patients with progressive melanoma brain metastases (MBM) there are no life prolonging treatments available to date. In this retrospective, single-center, case series, we show that combining the multi-kinase inhibitor regorafenib with standard-of-care BRAF/MEK inhibitors can have meaningful anti-tumor activity in melanoma patients with progressive MBM with an acceptable safety profile. These findings warrant further prospective investigations.

## 1. Introduction

Sixty to eighty percent of stage IV melanoma patients will develop melanoma brain metastases during the course of their disease (MBM; AJCC stage IV-M1d) [1]. Treating these remains a challenge, despite the availability of immune checkpoint blockade (ICB) and v-RAF murine sarcoma viral oncogene homolog B1 (BRAF)-/mitogen-activated protein kinase kinase (MEK) inhibitors (BRAF/MEKi), as a majority of patients with advanced melanoma still succumb to disease progression. Median overall survival (mOS) ranges from 26 to 72 months and the 5-year OS rates from 34 to 52% in several prospective phase 3 trials [2,3,4]. These trials, however, largely excluded stage IV-M1d melanoma patients, whose outcomes are worse. For instance, phase 2 clinical trials in patients with symptomatic MBM receiving ICB showed significantly lower response rates and a mOS ranging between 5.1 and 8.7 months [5,6]. Unlike ICB, the objective response rates (ORR) obtained with BRAF/MEKi, in *BRAF*^V600^-mutant AJCC stage IV-M1d melanoma are similar to patients without MBM, irrespective of whether the MBM are symptomatic. Nevertheless, survival outcomes are worse. In the phase 2 COMBI-MB trial, the mOS ranged from 10 to 24 months in asymptomatic patients and was only 11 months in symptomatic patients [7,8]. Finally, in the TRICOTEL trial, the combination of BRAF/MEKi and ICB in first line (atezolizumab, vemurafenib, cobimetinib) led to a mOS of 13.7 months in the total cohort of patients with MBM, and only 9 months in symptomatic patients [9]. These data emphasize the outstanding need for additional effective life-prolonging treatment options in stage IV-M1d melanoma. Enrollment into clinical trials is recommended for melanoma patients refractory to standard-of-care; however, patients with active MBM are often excluded from these [10].

In up to 90% of cutaneous melanomas, a somatic mutation leads to the hyperactivation of the mitogen-activated protein kinase (MAPK) pathway (RAS-RAF-MEK-ERK), resulting in enhanced cell survival, proliferation, invasion, and metastasis [11]. The most prevalent class I *BRAF*^V600^ mutation (35–50% of melanomas) gives rise to a constantly active monomer BRAF kinase, generating high levels of active phosphorylated ERK (p-ERK). These *BRAF*^V600^-mutant monomers are targeted by class I or monomer-selective BRAF inhibitors (BRAFi), approved for use in combination with MEK inhibitors (MEKi) in *BRAF*^V600^-mutant melanoma. Combinations of encorafenib/binimetinib (ENCO/BINI) and dabrafenib/trametinib (DAB/TRAM) yield ORRs of 64 and 68%, respectively [2,3]. However, long-lasting responses are often lacking due to the emergence of adaptive resistance. One proposed mechanism for adaptive resistance is the relief of ERK-induced negative feedback following the initial suppression of the ERK signal, following the effect of class I BRAFi. The loss of ERK-induced negative feedback releases the brake on cell surface receptor tyrosine kinase (RTK) signaling and allows for RAS-dependent RAF dimer formation and the subsequent reactivation of the MAPK pathway [12]. Using a class II, dimer-selective RAF inhibitor can overcome this resistance and subsequently halt the downstream reactivation of the MAPK pathway in preclinical models [12,13].

The *Neuroblastoma Ras Viral Oncogene Homolog*^Q61^ (*NRAS*^Q61^) mutation, the second most frequent MAPK pathway activating mutation in melanoma, leads to the canonical MAPK pathway activation through RAF dimerization as well as the activation of other parallel pathways, including the phosphoinositide 3-kinase (PI3K)–protein kinase B (AKT) and the mammalian target of rapamycin (mTOR) pathway [11,14,15]. To date, no targeted therapy is approved for *NRAS*^Q61^-mutant melanoma. Preclinical models suggest that combining RAF dimers with MEK inhibition may hold promise for *NRAS*-mutant melanoma [13,16]. A clinical phase Ib study with the combination of a RAF dimer inhibitor, naporafenib, and the MEKi trametinib showed objective responses in up to 46.7% of patients with a median progression-free survival (mPFS) of up to 5.52 months. However, 80% of patients experienced a rash, of which 23% were grade 3, requiring treatment interruption [17]. Our group has previously shown that MEKi-induced skin toxicity can be mitigated by adding a low-dose class I BRAFi [18].

Regorafenib (REGO) and sorafenib are currently the only approved RAF dimer inhibitors, with REGO being the most potent. In addition, REGO has kinase inhibitory effects against angiogenic, stromal, and oncogenic kinases [19,20]. It is approved for use in metastatic colorectal cancer, GIST, and hepatocellular carcinoma. Furthermore, there is evidence for REGO to enhance anti-tumor immunity (through VEGF-R and CSF-1R inhibition) in patients treated with ICB [19]. Recent preclinical findings highlight the potential of the combination of REGO with class I BRAFi and MEKi to overcome adaptive resistance to class I BRAFi [12].

Our group was the first to report on clinical experience with REGO for advanced, pretreated melanoma. In a retrospective cohort analysis, the continuous dosing of 40–80 mg REGO once daily demonstrated a manageable safety profile and hints at its activity as a monotherapy, but more pronounced efficacy was observed with the triple-targeted therapy combination of REGO + BRAF/MEKi [21]. In this single-center retrospective cohort study, we further analyzed the antitumor activity and safety of triple-targeted therapy (TTT): REGO + BRAF/MEKi, specifically in the AJCC stage IV-M1d*BRAF*- and *NRAS*-mutant melanoma patients, refractory to standard-of-care.

## 2. Materials and Methods

### 2.1. Study Design and Patient Selection

This retrospective single-center study was conducted at the Universitair Ziekenhuis Brussel (UZ Brussel), Brussels, Belgium. Prospectively identified patients aged 18 years and older with histologically confirmed unresectable AJCC-stage IV-M1d, *BRAF*- or *NRAS*-mutant (*BRAF*mut; *NRAS*mut resp.) melanoma who underwent REGO treatment in combination with BRAF/MEK inhibitors on a compassionate use basis between June 4th, 2021 and July 17th, 2023 were included. In all patients, progressive disease was documented under all available standard-of-care treatment options (ICB and BRAF/MEKi, if applicable) and patients were not eligible for prospective clinical trials. All patients were required to have an evaluable disease per the Response Evaluation Criteria in Solid Tumors, version 1.1 (RECIST v1.1), or the Response Assessment in Neuro-Oncology-Brain Metastases (RANO-BM) criteria. Therefore, patients needed to have undergone contrast-enhanced whole-body imaging (e.g., computed tomography (CT)) and/or the gadolinium contrast-enhanced magnetic resonance imaging of the brain (MRI) at baseline and as needed for response evaluation during follow-up [22,23]. Clinical records were reviewed for treatment disposition, safety, tumor response, and survival. As this is a retrospective study, the timing of response assessments, as well as treatment adjustments and interruptions were not prespecified. Database lock was on January 15th, 2024. The study protocol was approved by the Medical Ethics Committee of UZ Brussel/VUB (EC number: EC-2022-171). Patients alive at the time of the analysis provided written informed consent. The study was conducted in accordance with the Declaration of Helsinki and guidelines for Good Clinical Practice, as defined by the International Conference on Harmonization.

### 2.2. Endpoints

The first efficacy endpoint was overall ORR to the TTT (REGO + BRAF/MEKi) in *BRAF*-mutant melanoma and to REGO combined with full dose MEKi (plus low-dose BRAFi to mitigate MEKi-associated skin toxicity) in *NRAS*-mutant melanoma. Overall ORR is defined as the percentage of patients who showed a complete response (CR) or partial response (PR) at any time per RECIST v1.1 or per RANO-BM criteria in patients with MBM only. The second endpoint was intracranial and extracranial ORR (IC- and EC-ORR respectively), defined as the percentage of subjects with a CR or PR at any time, per RECIST v1.1 for extracranial lesions and per RANO-BM for MBM. Other endpoints were duration of response (DoR, overall, intracranial, and extracranial), defined as the time from response until first progression; time on triple-targeted therapy (ToT, defined as the time from the initiation of TTT until the last dose of TTT); the incidence and severity of treatment-related adverse events (TRAE), graded according to the Common Terminology Criteria of Adverse events (CTCAE), version 5.0; progression-free survival (PFS, time between treatment initiation and the earliest date of documented disease progression, clinical deterioration due to disease progression with treatment discontinuation, or death due to any cause); and overall survival (OS, defined as the time between treatment initiation and death due to any cause).

### 2.3. Statistical Analysis

Descriptive statistics were used to summarize and present the results. Demographic information was collected and summarized using frequency tables and proportions. For continuous variables, the median and standard deviation were calculated. Categorical variables, such as adverse events, were analyzed using frequency and percent distributions. Median PFS, OS, ToT, and DoR were estimated using the Kaplan–Meier method (SPSS Statistics version 28, IBM, Armonk, New York, NY, USA).

## 3. Results

### 3.1. Baseline Patient Characteristics

Twenty-three patients with stage IV-M1d, *BRAF*- or *NRAS*mut melanoma who underwent treatment with regorafenib in combination with BRAF/MEKi were identified. One patient was excluded from analysis due to a concurrent chronic lymphocytic leukemia being actively treated.

Among the 22 remaining patients (13 male, median age 52.7 y), 17 patients had a *BRAF*^V600^ mutation as an oncogenic driver mutation, one patient had a RAF fusion (PRKD1-BRAF fusion), and four had a *NRAS*^Q61^ mutation. For all analyses, the findings of the patient with the RAF fusion were combined with the *BRAF*^V600^-mutant patients (*BRAF*mut subgroup) because this patient received the same combination of REGO + full dose BRAF/MEKi as the *BRAF*^V600^-mutant patients. This RAF fusion is a class II RAF mutation acting as a constitutively active dimer with intermediate-to-high BRAF kinase activity, stimulating the MAPK pathway independently from upstream RAS activation [24].

All patients were pretreated with anti-PD-1 ICB, and 21 also received anti-CTLA-4 ICB. All *BRAF*mut patients progressed on BRAF/MEKi. The median number of prior systemic therapy lines was 3 (range 2–13). Nineteen patients (86%) had active progression in the brain at initiation of the TTT-regimen, and seventeen patients had five or more intracranial lesions. Sixteen patients (73%) had received prior intracranial treatment. Eleven patients (50%) were receiving corticosteroids (nine (41%) were receiving ≥ 32 mg methylprednisolone per day). Ten patients (45%) had an elevated baseline serum lactate dehydrogenase (LDH). The ECOG performance status (PS) was 0, 1, 2, or 3 in six (27%), six (27%), eight (36%), and two (9%) patients, respectively. In the seventeen *BRAF*^V600^-mutant patients, eight (47%) were previously rechallenged with BRAF/MEKi after at least a three-month interruption, thirteen (76%) were progressive on BRAF/MEKi at time of initiation of the TTT (REGO was added to the BRAF/MEKi), and only one patient had been off BRAF/MEKi for at least three months prior to initiation of the TTT-regimen (Table 1).

### 3.2. Treatment Disposition

REGO was administered orally, once daily (OD), and continuously without planned interruptions. It was initiated at 40 mg in 20 patients (91%) and at 80 mg in two (9%). Treatment with 40 mg of REGO was successfully increased to 40/80 mg on alternating days (AD) and 80 mg OD in three and seven patients out of twenty, respectively. Four patients (two *BRAF*^V600^mut) had received REGO as monotherapy (*n =* 3) or in combination with anti-PD-1 + anti-CTLA-4 ICB (*n =* 1) prior to the association of BRAF/MEKi.

**Figure 1:**
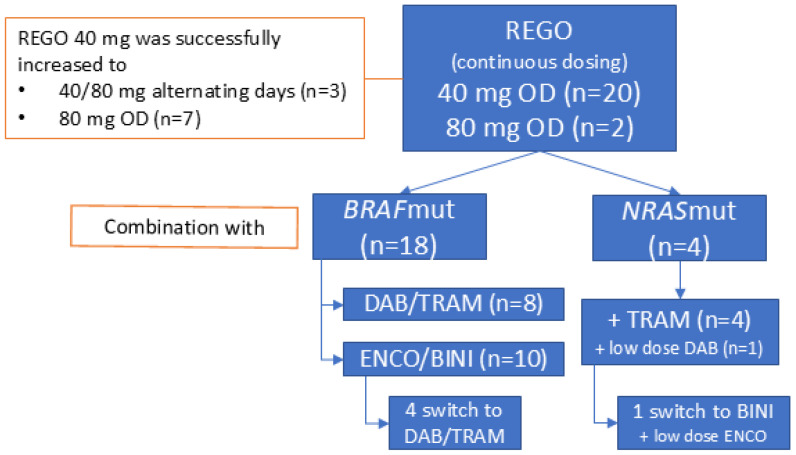
General overview of treatment disposition and triple-targeted therapy combinations used. Abbreviations: REGO—regorafenib; OD—once daily; DAB—dabrafenib; TRAM—trametinib; ENCO—encorafenib; BINI—binimetinib.

The 18 *BRAF*mut patients underwent combined REGO with ENCO/BINI (*n =* 10, 56%) or DAB/TRAM (*n =* 8, 44%) treatment. The starting doses of ENCO and BINI ranged between 150–450 mg OD and 15–45 mg twice a day (BID), respectively. The starting doses of DAB and TRAM ranged between 75 and 150 mg BID and between 1 and 2 mg OD, respectively. This was based on the dose the patient had sufficiently tolerated in the past or was receiving at the time of REGO association. The median time on TTT (ToT) was 7.6 weeks [95% CI 0–51 weeks]. In sixteen patients with at least six weeks ToT, eight (50%) received the full dose BRAF/MEKi with REGO for more than half of the treatment duration, and three and five patients received a lower dose MEKi with full dose or lower dose BRAFi, respectively, for more than half of the treatment duration. In four *BRAF*mut patients (22%) ENCO/BINI was replaced with DAB/TRAM, in three of whom this was due to a TRAE, possibly related to ENCO/BINI (incl. colonic ulcer, diarrhea, and rash) (Figure 1). In eight patients (44%), a temporary treatment interruption of all three drugs was needed because of TRAE. In five (28%), this interruption lasted longer than 7 days due to the insufficient recovery of the TRAE [range 9–15 days]. Four patients (22%) needed more than one treatment interruption. REGO, BRAFi, and MEKi were interrupted individually in four (22%), two (11%) and six (33%) patients, respectively. The dose of REGO, BRAFi, and MEKi was reduced in four (22%), four (22%) and seven (39%) patients, respectively, due to TRAE, including fever, rash, and diarrhea. The decision regarding which compound had to be reduced was at the discretion of the treating physician and was based on the type of TRAE, with the modification of the dosage of the drug assumed to be most likely responsible for the TRAE. For example, in case of acneiform dermatitis, the MEKi was reduced. In case of overlapping toxicities between the drugs, such as diarrhea, the dosages of all three drug were modified (e.g., reduced if a lower dose level was possible). No permanent treatment discontinuations due to toxicity were needed. At database lock, treatment was ongoing in two *BRAF*mut patients at 26 weeks and 46 weeks after initiation.

The four *NRAS*mut patients combined REGO (40 mg OD) with TRAM at 0.5–1 mg OD. The median time on treatment was 13.1 weeks [95% CI 10–16 weeks]. A low-dose BRAFi was associated in two patients to mitigate MEKi-induced skin-toxicity (acneiform dermatitis). In one patient, the association of ENCO 150 mg OD to BINI 30 mg BID led to the resolution of the MEKi-induced acneiform dermatitis. In the other patient, DAB 50 mg BID was associated to TRAM for the same acneiform dermatitis. In this patient, the effect was not evaluable because the patient died shortly after from infectious pneumonia (Figure 1). In three patients (75%), a treatment interruption was needed (longer than 7 days in two patients, including a patient interrupting treatment for 70 days to initiate a new line of treatment (temozolomide), after which the patient reinitiated REGO + TRAM beyond first progression for clinical benefit. One patient had to interrupt treatment more than once. One patient needed a dose reduction in REGO and TRAM for abdominal pain (80 to 40 mg and 1.5 to 0.5 mg, respectively). There were no permanent treatment interruptions. A detailed overview of the treatment disposition per individual patient is provided in Supplementary Table S1.

### 3.3. Safety

Twenty patients (90%) experienced at least one treatment-related adverse event (TRAE). The most frequently reported TRAEs were diarrhea, fatigue, abdominal pain, and acneiform rash/dermatitis. There were no grade 4 or 5 TRAEs. Ten patients (45%) experienced one or more reversible grade 3 TRAE, including arterial hypertension, maculo-papular rash, and duodenal perforation (resolving spontaneously after REGO interruption, while maintaining BRAF/MEKi). A summary of the TRAE is shown in Table 2 (complete overview in Supplementary Table S2).

### 3.4. Efficacy and Survival

**Figure 2:**
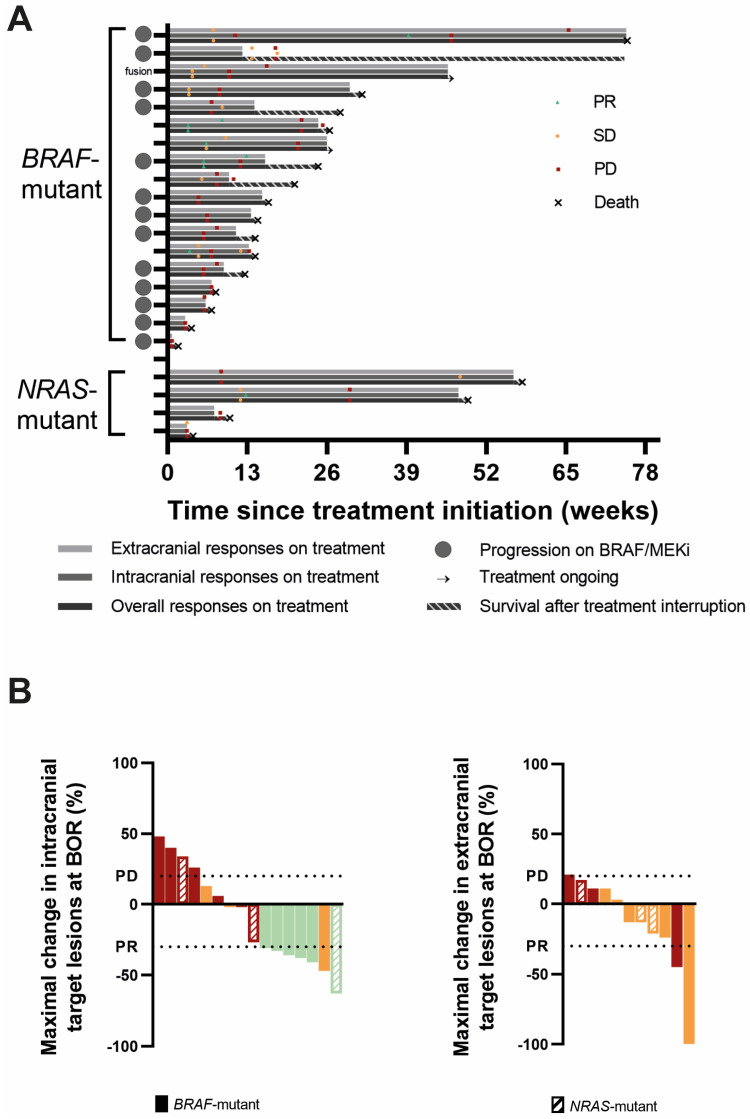
Responses to triple-targeted therapy. (**A**). Swimmer plots of all *BRAF*- and *NRAS*-mutant patients representing extracranial, intracranial, and overall responses, respectively, from top to bottom. The best objective responses (BORs) and moments of extracranial, intracranial, and overall progression are marked on the respective bars. The duration of survival after treatment interruption is shown as a striped bar. The gray circles on the Y-axis depict patients in whom REGO was associated following progression on BRAF/MEKi. (**B**). Waterfall plots of the maximal change in sum of target lesion diameters from baseline in the intracranial (**left**) and extracranial (**right**) target lesions in patients in whom the target lesions were evaluable for response and which changed in size. The color stands for the best objective response intracranially (**left**) and extracranially (**right**): red = progressive disease (PD); orange = stable disease (SD); and green = partial response (PR). The horizontal lines depict a change in size of the target lesions of at least +20% (PD) and −30% (PR) compared to baseline.

All 18 *BRAF*mut patients were evaluable for overall response at least one scan was performed of the intra- or extracranial lesions or there was clear information about neurological deterioration, considered as PD according to RANO-BM. The best objective response (BOR) was a partial response (PR) in two patients, stable disease (SD) in six patients, and progressive disease (PD) in ten patients, yielding an overall ORR and overall disease control rate (CR + PR + SD; overall DCR) of 11% and 44%, respectively. One PR patient and three SD patients were progressive on BRAF/MEKi at the time of REGO association. One SD was in the patient carrying the RAF-fusion. The DoR was 6.0 and 18.6 weeks. When evaluating intracranial responses, 17 of the 18 *BRAF*mut patients were response-evaluable. Three patients did not undergo a brain MRI during follow-up or at baseline. Of these, two had rapid neurological deterioration, considered as progressive according to RANO-BM. The third patient had extracranial progression, but did not experience neurological deterioration; therefore, this patient is considered non-response-evaluable for intracranial disease. Accordingly, the IC-BOR was PR in 5, SD in 5, and PD in 7 out of 17 patients, yielding an IC-ORR and IC-DCR of 29% and 59%, respectively, with a median IC-DoR of 7 weeks (range 3.6–22.0 w). Two patients with PR and three patients with SD were were progressive on BRAF/MEKi at time of REGO association. The *BRAF*mut patient without intracranial progression at the initiation of TTT remained free of intracranial progression. Finally, for extracranial disease, 12 *BRAF*mut patients were response-evaluable. Three patients were excluded for response evaluation because they exhibited no evidence of extracranial disease (NED) at baseline and remained free of extracranial disease during follow-up. Three other patients were excluded because no on-treatment scan was performed. In the remaining 12 patients, there were two PR (EC-DoR of 11.7 and 13 w), five SD, and five PD, yielding an EC-ORR and EC-DCR of 17% and 58%, respectively. Twelve *BRAF*mut patients had evaluable intra- and extracranial disease. In those patients, intra- and extracranial responses were concordant except in five patients: three patients had an intracranial PR and extracranial SD, and two had intracranial SD but extracranial progression (Table 3, Figure 2 and Figure S1). It is of note that in three *BRAF*^V600^ mutant patients, a new *NRAS*^Q61^ mutation was discovered at progression, either by using a tumor biopsy (*n =* 1) or plasma circulating tumor DNA (ctDNA, *n =* 2) analysis. In two of these, there was rapid clinical deterioration with a fatal outcome shortly after.

All four *NRAS*mut patients were response-evaluable for overall response with SD in one and PD in three patients, yielding an overall ORR of 0% and an overall DCR of 25%. When considering IC-BOR, there was a PR in one (DoR 36.3 w), SD in one, and PD in two patients (IC-ORR 25%; IC-DCR 50%). The patient without active intracranial disease at treatment initiation remained free of progression. When considering extracranial disease, three *NRAS*mut patients were evaluable for response. Baseline evaluation was not available in one patient. The EC-BOR were SD in two and PD in one patient. In the three patients that were response-evaluable for both intra- and extracranial disease, the responses were not concordant. The patient with an intracranial PR had extracranial SD, the patient with intracranial SD had extracranial PD, and finally, a patient with intracranial PD had extracranial SD (Table 3, Figure 2 and Figure S1).

**Figure 3:**
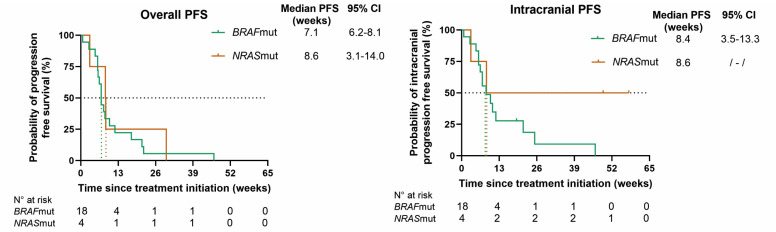

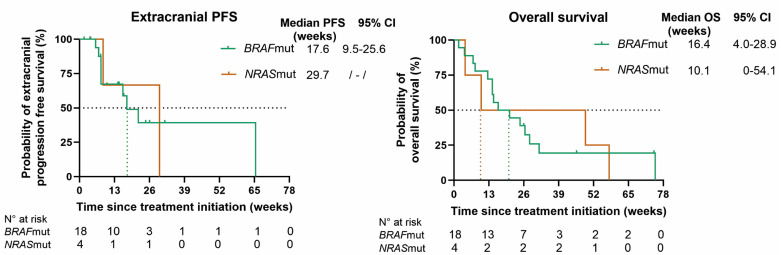
Kaplan–Meier curves for overall progression-free survival (PFS), intracranial PFS, extracranial PFS, and overall survival (OS) for BRAF (green)- and NRAS (orange)-mutant patients. Censored patients are shown as a vertical tick-mark. Abbreviations: 95% CI—95% confidence interval; N°—number.

At database lock, all patients had progressed extra- and/or intracranially. In *BRAF*mut patients, the mPFS rates for overall, intracranial, and extracranial disease, respectively, were 7.1 (95% CI 6.2–8.1), 8.4 (95% CI 3.5–13.3), and 17.6 (95% CI 9.5–25.6) weeks; in *NRAS*mut patients, the mPFS rates were 8.6 (95% CI 3.1–14.0), 8.6, and 29.7 weeks, respectively. The mOS rates in *BRAF*mut and *NRAS*mut patients were 16.4 (95% CI 4.0–28.9) and 10.1 weeks (95% CI 0–54.1) (Figure 3).

### 3.5. Treatment Beyond First Progression

Following the initial observations of rapid clinical deterioration due to accelerated disease progression upon treatment interruptions (cfr. case illustration) treatment beyond first progression was acceptable if clinical palliative benefit was suspected and discontinuing targeted therapy was considered to risk accelerated symptomatic disease progression. Thirteen *BRAF*mut patients (72%) continued treatment beyond first progression for a median duration of 6 weeks (range 2–36), and in two patients, treatment was ongoing at database lock. Strikingly, in two of the thirteen patients, new responses on a subsequent assessment were observed (one PR case according to RANO-BM, and one SD case with a reduction of 25% in tumor size of the target lesions compared to the brain MRI at progression (cfr case illustration)). Two of the four *NRAS*mut patients (50%) continued for an additional 18 and 48 weeks beyond first progression.

### 3.6. Case Illustration

**Figure 4:**
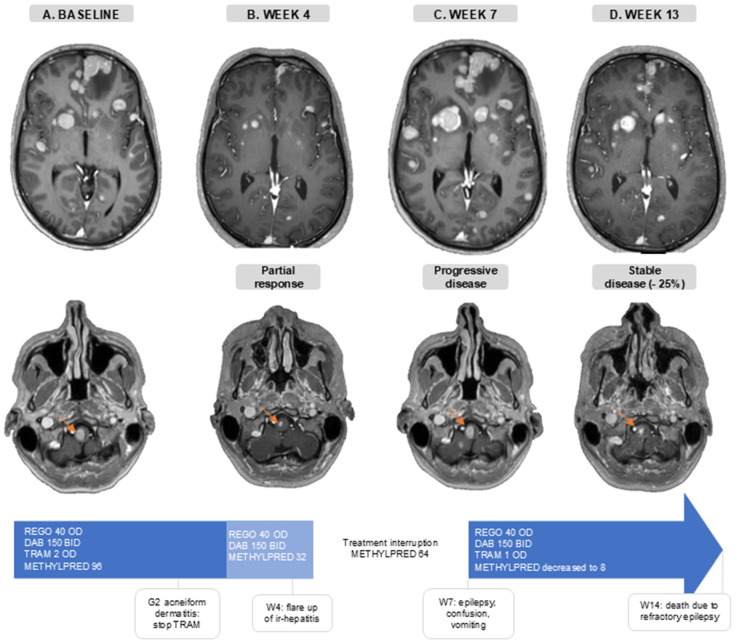
Case illustration. Axial slides of gadolinium-enhanced brain MRI. The orange arrow follows one brain metastasis in the brain stem. (**A**). Baseline, before triple-targeted therapy initiation. (**B**). After 4 weeks of triple-targeted therapy. (**C**). At 7 weeks after baseline MRI and 2 weeks after treatment interruption. (**D**). At 13 weeks after baseline scan and 6 weeks after triple-targeted therapy reinitiation. Doses are in mg. Abbreviations: BID—twice a day; DAB—dabrafenib; G—grade; ir-hepatitis—immune-related hepatitis; METHYLPRED—methylprednisolone; OD—once daily; REGO—regorafenib; TRAM—trametinib; W—week.

The disease of a 36-year-old patient progressed intra-and extracranially following anti-PD-1 ICB (nivolumab), dabrafenib/trametinib, and anti-PD-1 + anti-CTLA-4 (nivolumab + ipilimumab) treatment. At baseline, he presented with a generalized epileptic insult, due to active MBM, for which levetiracetam and methylprednisolone (96 mg/day) were initiated (Figure 4A). One week later, he initiated treatment of REGO 40 mg OD, DAB 150 mg BID, and TRAM 2 mg OD. During the first month he developed G2 acneiform dermatitis for which TRAM was interrupted. In the fourth week, he had intracranial PR and extracranial SD (−24% in extracranial target lesions), and methylprednisolone was decreased to 32 mg/day without the recurrence of neurological symptoms (Figure 4B). Around the same time, AST/ALT levels started rising, which was indicative of a flare up of a previous occurrence of immune-related hepatitis. Therefore, methylprednisolone was increased to 64 mg/day. When one week later (week 5), despite increasing corticosteroids, AST/ALT levels kept increasing (G3), the targeted therapy was interrupted and a second-line immune suppression with mycophenolate mofetil was associated. Thereafter, liver function tests normalized. In week 7, 13 days after the interruption of the targeted therapy, he was hospitalized with confusion, vomiting, and clinical signs of epilepsy. A brain MRI showed an important intracranial progression (Figure 4C). This illustrates the concept of rapid clinical deterioration at treatment interruption and the rational for treatment beyond first progression.. TTT was reinitiated: REGO 40 mg OD, DAB 150 mg BID, and TRAM 1 mg OD due to the prior acneiform dermatitis. During this second treatment course, methylprednisolone was successfully reduced to 8 mg/day, the patient regained independence in daily life and brain MRI showed a stable disease with a considerable decrease in the size of the target lesions (−25% compared to the MRI at first progression) (Figure 4D). On this regimen, he only experienced G1 acneiform dermatitis, and AST/ALT levels remained normal. Sadly, the renewed response was short-lived, and the patient died 14 weeks after initiating TTT.

## 4. Discussion

In this single-center retrospective case series, we conducted a comprehensive analysis of the efficacy and safety of regorafenib combined with BRAF/MEK inhibitors in twenty-two patients with stage IV-M1d, *BRAF*- or *NRAS*-mutant melanoma who were progressive after standard-of-care treatment with ICB and BRAF/MEK inhibitors (in case of *BRAF*^V600^-mutation). In *BRAF*-mutant patients, combination of REGO (a class II dimer-selective RAF inhibitor) with a class I monomer-selective BRAF inhibitor and a MEK inhibitor resulted in overall ORR and intracranial ORR of 11% and 29%, respectively, with an encouraging overall DCR of 44% and IC-DCR of 59%. In the *NRAS*^Q61^-mutant patients, REGO was combined with a full-dose MEK inhibitor (and if needed a low-dose *BRAF* inhibitor, to mitigate MEKi-induced skin toxicity). In this group, we observed overall SD in one patient and one intracranial PR and SD each.

The rationale for this TTT-combination stems from several preclinical models of solid tumors harboring MAPK pathway activating driver mutations. For instance, in *BRAF*mut melanoma cell lines and xenograft models with a relative insensitivity to RAF monomer-selective inhibitors, combining REGO + DAB/TAM overcomes adaptive resistance [12]. When considering non-*BRAF*^V600^-mutant tumors with other MAPK pathway activating driver mutations (e.g., *NRAS*, *NF1*, *BRAF*^non-V600^), the group of Hong et al. showed that combining a MEKi with a dimer-specific RAFi can prevent and overcome adaptive MEKi resistance in vitro and in vivo [13]. Several preclinical models also point to a possible synergistic effect of a pan-RAFi (including RAF dimer inhibition) and a MEKi on non-*BRAF*^V600^-mutant tumors (e.g., *NRAS*) as compared to MEKi treatment alone [16,25].

Our group is the first to confirm these preclinical findings in humans and to report on the efficacy of these combination strategies. Our first retrospective analysis on the compassionate use of REGO as a monotherapy or combined with other therapies (ICB or targeted therapies) in advanced melanoma patients showed the most pronounced responses when combining REGO + BRAF/MEKi [21]. In the case series reported here, we specifically focus on patients with MBM, who exclusively received triple-targeted therapy of REGO + BRAF/MEKi in *BRAF*mut melanoma and REGO + MEKi in *NRAS*mut melanoma. To the best of our knowledge, there is only one case report of a patient receiving REGO + DAB/TRAM: a stage IV advanced *BRAF*^V600^-mutant colorectal cancer patient who experienced a strong decrease in CEA-levels during a treatment course of 8 months [12].

Treating patients with MBM remains challenging. Prospective studies with ICB and BRAF/MEKi show comparable response rates in patients with asymptomatic melanoma brain metastasis as compared to patients without CNS involvement; however, once patients become symptomatic or require steroids, ICB becomes less performant [5,6,7,9,26]. The addition of upfront local treatment, such as stereotactic radiation, and treatment with ICB as first-line and BRAF/MEKi as second-line therapy can result in a favorable OS in this population [27]. However, once patients have intracranial progression beyond these treatment options, no treatment has shown to improve OS. A limited number of studies is ongoing in stage IV-M1d melanoma patients who have progressive disease past standard-of-care options, including studies using TIL (NCT05640193) or other targeted therapy regimens (NCT06194929). Nevertheless, patients with active and/or symptomatic MBM are generally excluded. Therefore, this case series represents a unique real-world dataset. We demonstrate intracranial tumor responses to REGO + BRAF/MEKi in *BRAF*^V600^-mutant melanoma patients with active and symptomatic MBM that were pretreated systemically as well as locally in a number of patients. Some of these responses lasted for more than three months.

When considering *NRAS*^Q61^-mutant melanoma, previous trials with binimetinib and the combination of trametinib and the pan-RAFi naporafenib have shown encouraging results in terms of response. However, binimetinib did not improve OS compared to dacarbazine in the NEMO trial, and naporafenib + trametinib was explored in a phase Ib trial with only 30 patients [17,28]. Additionally, in both trials, MEKi-related toxicity was an issue; for instance, 25% of the patients in the binimetinib group of the NEMO trial permanently discontinued treatment due to AEs [28]. In our cohort, MEKi-induced toxicity was successfully managed with the addition of a low-dose BRAFi, as shown previously [18,29].

While the concept of renewed responses upon rechallenge with BRAF/MEKi is known in melanoma [30,31], this does not explain all observed responses or stabilization of disease seen in this case series. For instance, 13 *BRAF*^V600^-mutant patients progressed on BRAF/MEKi at the time of REGO association. Two of these achieved IC-PR and three IC-SD, clearly illustrating the response to the TTT-regimen. Additionally, more than half of the *BRAF*^V600^-mutant patients had already undergone a prior rechallenge with BRAF/MEKi in the course of their previous treatment lines.

Our experience points towards a meaningful short-term palliative benefit from treatment beyond progression in selected patients, considering observations of accelerated symptomatic disease progression upon treatment interruptions, as illustrated with a case study. Surprisingly, we also observed a deepened response when treatment was continued beyond progression in one patient. We therefore advocate that treatment beyond first progression should be considered as a short-term palliative strategy in all patients progressive on targeted therapy regimens who are considered to derive a continued clinical benefit.

Resistance mechanisms of this novel TTT-regimen remain to be elucidated. In three *BRAF*^V600^-mutant patients, a new *NRAS*^Q61^ mutation emerged at progression. This is likely an acquired resistance mechanism that has previously been reported to be a MAPK pathway-reactivating resistance mechanism, found in approximately 18% *BRAF*^V600^-mutant melanoma progressive on standard-of-care BRAFi therapy [32,33]. Further prospective evaluation is needed to determine whether this mechanism of resistance is more common when using the TTT-combination.

Overall, the combination of REGO + BRAF/MEKi has a manageable safety profile. While grade 3 TRAEs occurred in 45% of the patients, all were reversible and self-limiting with treatment interruption. The adverse events specific for REGO such as hand–foot skin reaction, diarrhea, or hypertension were less frequent when comparing this series to the phase 3 trials that led to the approval of REGO in metastatic colorectal cancer, GIST, and hepatocellular carcinoma. For instance, hand–foot-skin reaction was only found in 18% of the patients in this case series as opposed to 47–56% in the phase 3 trials [34,35,36]. One possible explanation is the continuous dosing schedule of 40–80 mg OD used in our patients, as compared to the classical dosing schedule of 160 mg OD in 21/28 day cycles in the abovementioned phase 3 trials. Accumulating toxicities with the classical dosing are known to be treatment limiting and dose modifications are common [35]. The efficacy and safety of a lower dose of REGO (100 mg daily, continuous dosing) has been prospectively evaluated in GIST and showed fewer grade 3 toxicities with a comparable disease control rate and PFS [37]. Another explanation stems from a *BRAF*^V600^-mutant melanoma xenograft model in which the mice were exposed to REGO + TRAM and experienced weight loss, which suggested accumulating toxicities. The mice receiving the triple combination REGO + DAB/TRAM showed no evidence of weight loss [12]. This supports the rationale that in normal, healthy cells, the combination of the class I monomer-selective and class II dimer-selective RAF inhibitors, such as DAB and REGO, respectively, have an opposing effect on MAPK signaling (paradoxical activation by class I and suppression by class II RAF inhibitors) [12,38].

### Limitations and Future Perspectives

The retrospective nature of this case series comes with inherent limitations, such as the use of different dose schedules and adjustments, treatment interruptions not being uniform, and imaging not being performed to a prespecified schedule and, therefore, not all patients being response-evaluable for extra-and intracranial disease separately. However, it remains a unique dataset representing a real-world situation in a population that was heavily pretreated and without access to prospective clinical trials. Furthermore, as the patients were prospectively identified, follow-up was harmonized, response evaluations were performed systematically, and data concerning safety were registered with care. Overall, the results of regorafenib combined with BRAF/MEKi in a heavily pretreated and vulnerable patient population are promising and have led to the initiation of an ongoing prospective phase 2 clinical trial RegoMel (clinical trials.gov ID NCT05370807). Following a cohort in pretreated melanoma where regorafenib was tested as a monotherapy, two additional cohorts were added to the trial to test the TTT-regimen in advanced, pretreated *BRAF*^V600^-melanoma, including patients with active melanoma brain metastases. This will allow for a standardized dosing approach and a robust indication of the clinical efficacy. It will also allow for the exploration of the predictive biomarkers of response to therapy, such as total metabolic tumor volume, the integrated PET/MRI evaluation of brain metastases, or circulating tumor DNA (ctDNA).

## 5. Conclusions

In this case series, in heavily pre-treated melanoma patients with refractory brain metastases, triple-targeted therapy with regorafenib and BRAF/MEKi in *BRAF*-mutant patients and regorafenib with MEKi (+low-dose BRAFi) in *NRAS*-mutant patients shows a promising anti-tumor activity signal with an acceptable safety profile. In *BRAF*mut patients, there was an overall ORR and IC-ORR of 11 and 29%, respectively, and an overall DCR and IC-DCR of 44 and 59%, respectively. In *NRAS*mut patients, the overall ORR and IC-ORR were 0 and 25%, and overall DCR and IC-DCR were 25 and 50%, respectively. The median OS was 16.4 and 10.1 weeks, respectively, in *BRAF*mut and *NRAS*mut patients. These findings warrant further prospective exploration, especially in *BRAF*^V600^-mutant melanoma in order to better understand the efficacy and possible resistance mechanisms of this triple-targeted therapy regimen.

## Figures and Tables

**Table 1:** Baseline patient characteristics.

Baseline Patient Characteristics *n* = 22			
** Age, Years **		**Corticosteroids at Treatment Initiation, No. (%)**	
Median	52.7	None	11 (50%)
Range	29–74	<32 mg methylprednisolone or equivalent	2 (9%)
** Gender, No. (%) **		≥32 mg methylprednisolone or equivalent	9 (41%)
Male	13 (59%)	**Type of prior local intracranial treatment, No. (%)**	
Female	9 (41%)	None	6 (27%)
** ECOG performance-status score, No. (%) **		Surgery	14 (64%)
0	6 (27%)	SRS/SRT	8 (36%)
1	6 (27%)	WBRT	2 (9%)
2	8 (36%)	**Type of prior systemic therapy, No. (%)**	
3	2 (9%)	ICB *	22 (100%)
** Ethnicity, No. (%) **		BRAF/MEKi	21 (95%)
Caucasian	22 (100%)	Chemotherapy	4 (18%)
** Driver mutation, No. (%) **		T-VEC	2 (9%)
*BRAF* ^V600E/D ^	17 (77%)	**Prior lines of systemic therapy, No. (%)**	
* NRAS* ^Q61R/K ^	4 (18%)	2	2 (9%)
* PRKD1-BRAF * fusion	1 (5%)	3	11 (54%)
** Baseline LDH, No. (%) **		4	3 (14%)
≤ULN	11 (50%)	5	1 (5%)
>ULN	10 (45%)	≥6	5 (23%)
Unknown	1 (5%)	***BRAF*****^V600 ^ -mutant patients only** ***n*** = **17**	
** Intracranial disease progression at treatment initiation **		**Previous rechallenge BRAF/MEKi °, No. (%)**	
Yes	19 (86%)	Yes	8 (47%)
No	2 (9%)	No	9 (53%)
Unknown	1 (5%)	**Progression on BRAF/MEKi at treatment initiation, No. (%)**	
** Number of brain metastases, No. (%) **		Yes	13 (76%)
1	2 (9%)	No	4 (24%)
2	2 (9%)		
3	0 (0%)		
4	1 (5%)		
≥5	17 (77%)		

* ICB (immune checkpoint blockade) with anti-PD-1 monoclonal antibodies and/or anti-CTLA-4 monoclonal antibodies. ° Rechallenge defined as reinitiation of BRAF/MEKi after at least three months of interruption of these drugs. Abbreviations: BRAF/MEKi: BRAF/MEK inhibitors; ICB: immune checkpoint blockade; LDH: lactate dehydrogenase; ULN: upper limit of normal; SRS: stereotactic radiosurgery; SRT: stereotactic radiotherapy; WBRT: whole brain radiotherapy.

**Table 2:** Treatment-related adverse events.

Treatment-Related Adverse Event *n* = 22	Any Grade n (%)	Grade 3 n (%)
Any TRAE	20 (91%)	10 (45%)
Diarrhea	12 (55%)	1 (5%)
Fatigue	10 (45%)	
Abdominal pain	9 (45%)	
Rash acneiform	9 (41%)	
AST/ALT increase	7 (32%)	1 (5%)
Rash maculo-papular	6 (27%)	3 (14%)
Fever	5 (23%)	
Hypophosphatemia	5 (23%)	
Arterial hypertension	4 (18%)	4 (18%)
Anemia	4 (18%)	1 (5%)
Anorexia	4 (18%)	1 (5%)
Hand–foot skin reaction	4 (18%)	
Platelet count decreased	4 (18%)	
CPK increased	3 (14%)	1 (5%)
Alopecia	3 (14%)	
Constipation	3 (14%)	
Dry skin	3 (14%)	
Headache	3 (14%)	
Nausea	3 (14%)	
Skin ulceration	3 (14%)	
Colonic hemorrhage	1 (5%)	1 (5%)
CRP increased	1 (5%)	1 (5%)
Duodenal perforation	1 (5%)	1 (5%)

Shown are treatment-related adverse events of any grade reported in more than 10% of patients and adverse events of grade 3. Adverse events were graded according to the National Cancer Institute Common Terminology Criteria for Adverse Events, version 5.0. Abbreviations: AST—aspartate transaminase; ALT—alanine transaminase; CPK—creatine phosphokinase; CRP—C-reactive protein.

**Table 3:** Tumor responses according to RECIST v1.1 and/or RANO-BM.

	Best Overall Response	Best Intracranial Response	Best Extracranial Response
***BRAF*** **mut, No. (%) **	***n *= 18**	*n *= 17 *	*n *= 12 **
Partial response	2 (11%)	5 (29%)	2 (17%)
Stable disease	6 (33%)	5 (29%)	5 (42%)
Progressive disease	10 (56%)	7 (41%)	5 (42%)
** ORR ** (%)	11%	29%	17%
** DCR ** (%)	44%	59%	58%
***NRAS*** **mut, No. (%) **	***n *= 4**	***n* = 4**	*n *= 3 ***
Partial response	0	1 (25%)	0
Stable disease	1 (25%)	1 (25%)	2 (67%)
Progressive disease	3 (75%)	2 (50%)	1 (33%)
** ORR ** (%)	0%	25%	0%
** DCR ** (%)	25%	50%	66%

* In one patient, the intracranial response according to RANO-BM was unknown. ** In three patients, the extracranial response according to RECIST v1.1 was unknown, and in three patients, there was no evidence of extracranial disease at baseline as well as during follow-up. *** In one patient, the extracranial response according to RECIST v1.1 was unknown. Abbreviations: *BRAF*mut—*BRAF*-mutant; *NRAS*mut—*NRAS*^Q61^-mutant; ORR—overall response rate; DCR—disease control rate.

## Data Availability

The dataset generated for this case series contains confidential patient information but can be made available upon request to interested researchers.

## Supplementary Materials

The following supporting information can be downloaded at: https://www.mdpi.com/article/10.3390/cancers16234083/s1. Figure S1: Change in tumor size over time in response to TTT; Table S1: Treatment disposition in individual patients; Table S2: Complete list of treatment-related adverse events.
