# Supplementary material for: Regorafenib Combined with BRAF/MEK Inhibitors for the Treatment of Refractory Melanoma Brain Metastases
Source: Cancers (Basel). 2024 Dec 5;16(23):4083. doi: 10.3390/cancers16234083 (PMC11640054; doi:10.3390/cancers16234083)
Supplement: Supplementary file 1 [file cancers-16-04083-s001.zip › Supplementary figure.pdf]

Supplementary figure

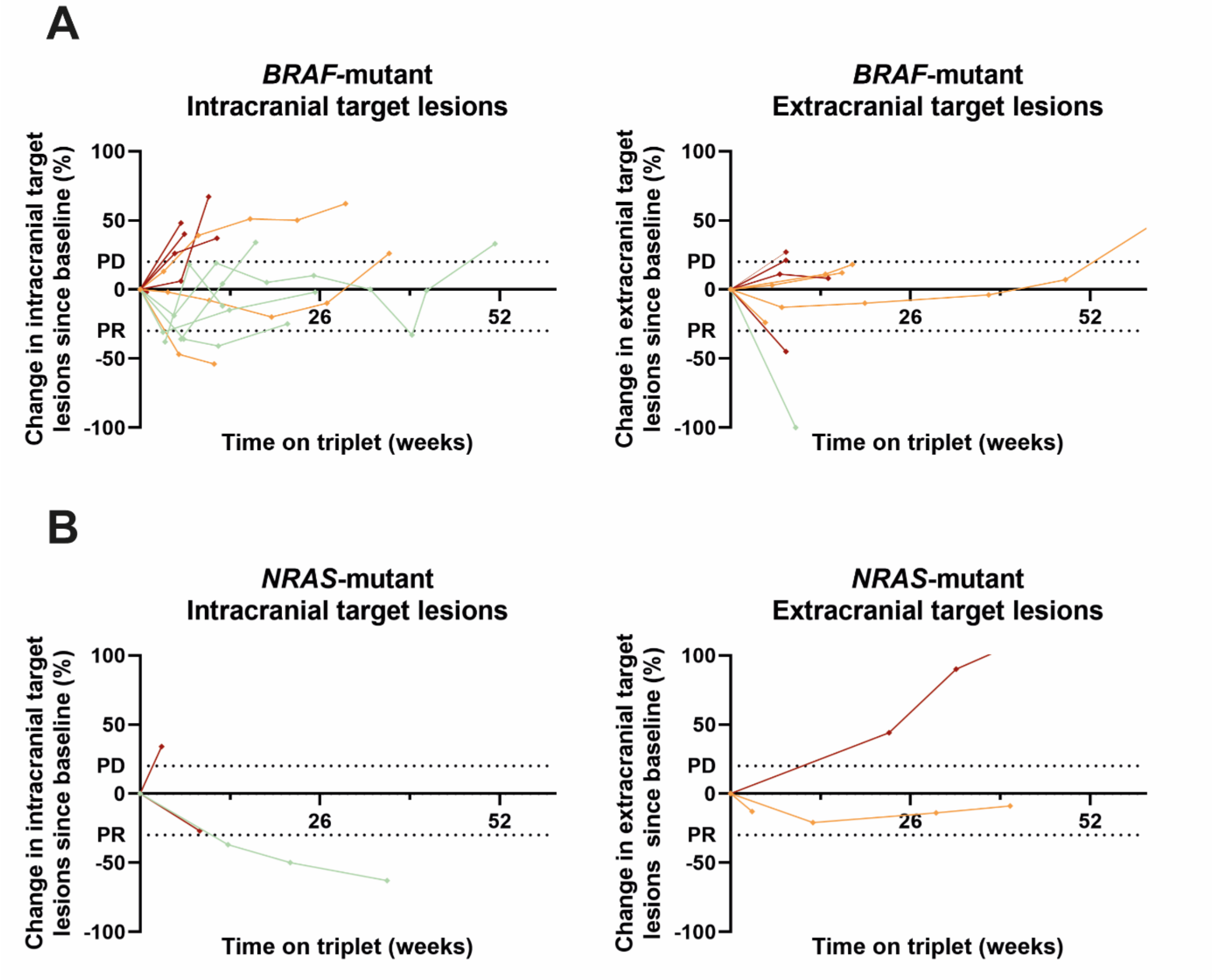

Supplementary figure S1. Change in tumor size over time in response to TTT. These spider plots show the changes in tumor size from baseline. A. shows changes in intracranial (left) and extracranial (right) target lesions in BRAF-mutant patients. B. shows changes in intracranial (left) and extracranial (right) target lesions in NRAS-mutant patients. The green line represents partial response, orange line represents stable disease, and the red line represents PD as best objective response. The horizontal line labeled PD depicts a change in size of at least + 20% and PR a change in size of at least – 30% compared to baseline.
