# Supplementary material for: Regorafenib Combined with BRAF/MEK Inhibitors for the Treatment of Refractory Melanoma Brain Metastases
Source: Cancers (Basel). 2024 Dec 5;16(23):4083. doi: 10.3390/cancers16234083 (PMC11640054; doi:10.3390/cancers16234083)
Supplement: Supplementary file 1 [file cancers-16-04083-s001.zip › Supplementary tables.pdf]

Supplementary tables

Supplementary table S1. Treatment disposition in individual patients

	Regorafenib (mg)	BRAF-inhibitor (type, mg)	MEK-inhibitor (type, mg)	Other treatment	Duration (weeks)
***BRAF*-mutant patients**					
RRM13<br>ToT: 74.9w<br>PFS: 46.3w	40 OD	DAB 75 BID	TRAM 1 OD		1,0
	40 OD	DAB 150 BID	TRAM 1 OD		0,9
	Treatment interruption				0,7
	40 OD	DAB 75 BID	TRAM 1 OD		0,6
	40 OD				1,3
	80 OD	DAB 50 BID	TRAM 0.5 OD		1,3
	Treatment interruption				1,3
	80 OD				1,0
	80 OD		TRAM 1 OD		1,9
	80 OD	DAB 50 BID	TRAM 1 OD		1,0
	80 OD	DAB 50 BID			0,7
	80 OD	DAB 75 BID			0,7
	80 OD	DAB 75 BID	TRAM 0.5 OD		0,6
	80 OD	DAB 150 BID	TRAM 0.5 OD		5,1
	80 OD	DAB 150 BID	TRAM 1 OD		11,7
		DAB 150 BID	TRAM 1 OD		3,9
	40 OD	DAB 150 BID	TRAM 1 OD		10,9
	80 OD	DAB 150 BID	TRAM 1 OD		7,9
	80 OD	DAB 150 BID	TRAM 1 OD	TMZ	7,9
		DAB 150 BID	TRAM 2 OD		12,4
RRM35<br>ToT: 45.7w<br>PFS: 10w	40 OD	ENCO 450 OD	BINI 45 BID		2,0
	80 OD	ENCO 450 OD	BINI 45 BID		1,9
	80 OD	ENCO 300 OD	BINI 30 BID		1,9
	80 OD	DAB 150 BID	TRAM 2 OD		8,0
	80 OD	DAB 150 BID			1,0
	80 OD	DAB 150 BID	TRAM 2 OD		30,3
RRM33<br>ToT: 29.7w<br>PFS: 8.4w	40 OD	DAB 150 BID	TRAM 2 OD		1,4
	Treatment interruption				1,3
	40 OD	DAB 150 BID	TRAM 1 OD		1,9
	40 OD	DAB 150 BID	TRAM 0.5 OD		3,9
	40/80 AD	DAB 150 BID	TRAM 0.5 OD		1,9
	40/80 AD	DAB 150 BID			0,9
	40/80 AD	DAB 150 BID	TRAM 0.5 OD		0,9
	40/80 AD	DAB 150 BID	TRAM 1 OD		9,6
		DAB 150 BID	TRAM 1 OD		1,0

	40/80 AD	DAB 150 BID	TRAM 1 OD		6,7
		Treatment interruption			1,9
RRM44<br>ToT: 26.0w<br>PFS: 21.3w	80 OD	ENCO 450 OD	BINI 45 BID		1,1
		Treatment interruption			2,1
		ENCO 450 OD	BINI 45 BID		0,9
		DAB 100 BID	TRAM 1 OD		0,9
	40 OD	DAB 100 BID	TRAM 1 OD		0,7
	40 OD				0,4
	40 OD	ENCO 450 OD			0,3
	40 OD	DAB 100 BID			0,9
	40 OD	DAB 100 BID		DAB/TRAM 5/7 DAYS	1,0
	40 OD	DAB 100 BID	TRAM 0.5 OD		1,9
	40 OD	DAB 100 BID	TRAM 0.5 OD		9,9
	80 OD	DAB 100 BID	TRAM 0.5 OD		4,6
RRM9<br>ToT: 24.6w<br>PFS: 21.9w	80 OD	ENCO 150 OD	BINI 15 BID		19,9
	80 OD	ENCO 300 OD	BINI 15 BID		4,7
RRM19<br>ToT: 15.9w<br>PFS: 11.9w	40 OD	ENCO 300 OD	BINI 30 BID		2,6
		ENCO 300 OD	BINI 30 BID		0,9
	40 OD	ENCO 300 OD	BINI 30 BID		2,3
	40 OD	ENCO 300 OD	BINI 30 BID		9,7
RRM31<br>ToT: 15.4w<br>PFS: 5w	40 OD	ENCO 300 OD	BINI 30 BID		3,3
		Treatment interruption			0,6
	40 OD	ENCO 300 OD	BINI 30 BID		4,0
		Treatment interruption			1,0
	40 OD	ENCO 300 OD	BINI 30 BID		4,0
		Treatment interruption			0,7
	40 OD	DAB 100 BID	TRAM 1 OD		1,0
RRM28<br>ToT: 14.1w<br>PFS: 7.1w	40 OD	ENCO 450 OD	BINI 45 BID		2,3
	80 OD	ENCO 450 OD	BINI 45 BID		2,1
	40 OD	ENCO 450 OD	BINI 45 BID		2,1
		Treatment interruption			2,1
		ENCO 450 OD	BINI 45 BID		0,4
	40 OD	ENCO 450 OD	BINI 45 BID		1,0
	40/80 AD	ENCO 450 OD	BINI 45 BID		3,3
RRM42<br>ToT: 13.6w<br>PFS: 6.4w	40 OD	DAB 100 BID	TRAM 1.5 OD		0,7
	80 OD	DAB 100 BID	TRAM 1.5 OD		4,0
	80 OD	DAB 100 BID			3,4
	80 OD	DAB 100 BID	TRAM 0.5 OD		5,0
RRM30<br>ToT: 13.1w<br>PFS: 7.1w	40 OD	DAB 150 BID	TRAM 2 OD		4,0
	40 OD	DAB 150 BID			0,9
		Treatment interruption			1,7
	40 OD	DAB 150 BID	TRAM 1 OD		6,1

RRM1<br>ToT: 12.1w<br>PFS: 17.6w	40 OD	ENCO 450 OD	BINI 45 BID		12,1
RRM29<br>ToT: 11.1w<br>PFS: 5.9w	40 OD	DAB 150 BID	TRAM 2 OD		7,9
	80 OD	DAB 150 BID	TRAM 2 OD		2,0
	40/80 AD	DAB 150 BID	TRAM 2 OD		1.0
RRM41<br>ToT: 10.0w<br>PFS: 8.0w	40 OD	ENCO 450 OD	BINI 45 BID		0,6
	Treatment interruption				0,1
	40 OD	ENCO 450 OD	BINI 45 BID		3,7
	40 OD	ENCO 450 OD	BINI 45 BID		0,6
	80 OD	ENCO 450 OD	BINI 45 BID		0,6
	Treatment interruption				0,3
	40 OD	ENCO 450 OD	BINI 45 BID		3,1
RRM34<br>ToT: 9.1w<br>PFS: 5.9w	40 OD	ENCO 450 OD	BINI 45 BID		3,0
	Treatment interruption				0,7
	40 OD	ENCO 300 OD	BINI 30 BID		4,0
	40 OD	DAB 150 BID	TRAM 2 OD		1,0
RRM32<br>ToT: 7.1w<br>PFS: 7.1w	40	DAB 150 BID	TRAM 2 OD		7,1
RRM38<br>ToT: 6.0w<br>PFS: 6.0w	40 OD	ENCO 450 OD	BINI 45 BID		1,9
	80 OD	ENCO 450 OD	BINI 45 BID		4,1
RRM37<br>ToT: 2.9w<br>PFS: 2.9w	40 OD	DAB 150 BID	TRAM 2 OD		1,0
	80 OD	DAB 150 BID	TRAM 2 OD		2,7
RRM39<br>ToT: 0.7w<br>PFS: 0.7w	40	DAB 150 BID	TRAM 2 OD		0,7
***NRAS*-mutant patients**					
RRM10<br>ToT: 56.4w<br>PFS: 8.7w	40 OD		TRAM 0.5 OD		1,9
	40 OD		TRAM 1 OD		4,4
	40/80 AD		TRAM 1 OD		3,4
	80 OD		TRAM 1 OD		12,9
	Other treatment			TMZ	7,3
	80 OD		TRAM 1 OD		2,0
	80 OD		TRAM 1.5 OD		1,9
	80 OD		TRAM 1.5 OD		1,6
	40 OD		TRAM 0.5 OD		12,1
	40/80 AD		TRAM 0.5 OD		2,9

	40/80 AD		TRAM 1 OD	2,4
RRM2<br>ToT: 47.4w<br>PFS: 29.7w	40 OD		TRAM 0.5 OD	5,9
	40 OD		TRAM 1 OD	15,9
		Treatment interruption		2,6
	40 OD			0,9
	40 OD		BINI 15 BID	1,6
		Treatment interruption		2,6
	40 OD		BINI 15 BID	0,9
	40 OD		BINI 30 BID	5,0
		Treatment interruption		0,7
	40 OD	ENCO 150 OD	BINI 30 BID	3,6
	40/80 AD		BINI 30 BID	6,9
RRM15<br>ToT: 7.6w<br>PFS: 8.6w	40 OD		TRAM 1 OD	3,9
		Treatment interruption		0,9
	80 OD			1,4
	80 OD	DAB 50 BID	TRAM 1 OD	1,0
RRM11<br>ToT: 3.1w<br>PFS: 3.1w	40 OD		TRAM 1 OD	3,1

Table legend. Shown is the treatment disposition with dose and duration of regorafenib (REGO), BRAF-inhibitors (dabrafenib or encorafib), MEK-inhibitors (trametinib or binimetinib) and other treatments as well as treatment interruptions for each individual patient. RRMXX signifies the study code. The BRAF-mutant patient harbouring the RAF-fusion is patient RRM35. Abbreviations: AD: alternating days; BID: twice a day; BINI: binimetinib; DAB: dabrafenib; ENCO: encorafenib; PFS: progression free survival; OD: once daily; TMZ: temozolomide; ToT: time on treatment; TRAM: trametinib; w: weeks.

Supplementary table S2. Complete list of treatment related adverse events

**Treatment related adverse event n=22**	**Any grade n (%)**	**Grade 3 n (%)**
Diarrhea	12 (55%)	1 (5%)
Fatigue	10 (45%)	
Abdominal pain	9 (41%)	
Rash acneiform	9 (41%)	
AST/ALT increase	7 (32%)	1 (5%)
Rash maculo-papular	6 (27%)	3 (14%)
Fever	5 (23%)	
Hypophosphatemia	5 (23%)	
Arterial hypertension	4 (18%)	4 (18%)
Anemia	4 (18%)	1 (5%)
Anorexia	4 (18%)	1 (5%)
Palmar-plantar erythrodysesthesia syndrome	4 (18%)	
Platelet count decreased	4 (18%)	
CPK increased	3 (14%)	1 (5%)

Alopecia	3 (14%)	
Constipation	3 (14%)	
Dry skin	3 (14%)	
Headache	3 (14%)	
Nausea	3 (14%)	
Skin ulceration	3 (14%)	
Blood lactate dehydrogenase increase	2 (9%)	
Cardiac troponin I increased	2 (9%)	
Creatinine increase	2 (9%)	
Creatinine increased	2 (9%)	
Hypocalcemia	2 (9%)	
Hypomagnesemia	2 (9%)	
Muscle weakness lower limb	2 (9%)	
Colonic hemorrhage	1 (5%)	1 (5%)
CRP increased	1 (5%)	1 (5%)
Duodenal perforation	1 (5%)	1 (5%)
Amenorrhea	1 (5%)	
Back pain	1 (5%)	
Chills	1 (5%)	
Colonic ulcer	1 (5%)	
Confusion	1 (5%)	
Conjunctival bleeding	1 (5%)	
Dry mouth	1 (5%)	
Duodenal ulcer	1 (5%)	
Dyspepsia	1 (5%)	
Dyspnea	1 (5%)	
Ejection fraction decreased	1 (5%)	
Eosinophilia	1 (5%)	
Flu like symptoms	1 (5%)	
Gastric ulcer	1 (5%)	
Hematoma	1 (5%)	
Hoarseness	1 (5%)	
Hyperhidrosis	1 (5%)	
Hypokalemia	1 (5%)	
Hyponatremia	1 (5%)	
Insomnia	1 (5%)	
Lymphocyte count decreased	1 (5%)	
Menorrhagia	1 (5%)	
Muscle cramps	1 (5%)	
Myalgia	1 (5%)	
Nystagmus	1 (5%)	
Oral dysesthesia	1 (5%)	
Oral mucositis	1 (5%)	
Paronychia	1 (5%)	
Presyncope	1 (5%)	
Soft tissue infection	1 (5%)	
Sore throat	1 (5%)	
Thromboembolic event	1 (5%)	
Weight loss	1 (5%)	

White blood cell decreased	1 (5%)

Table legend. Shown are all treatment related adverse events. Adverse events were graded according to the National Cancer Institute Common Terminology Criteria for Adverse Events, version 5.0. Abbreviations: AST: aspartate transaminase; ALT: alanine transaminase; CPK: creatine phosphokinase; CRP: C-reactive protein
